# The Association of PTSD Symptom Severity With Localized Hippocampus and Amygdala Abnormalities

**DOI:** 10.1177/2470547017724069

**Published:** 2017-08-08

**Authors:** Teddy J. Akiki, Christopher L. Averill, Kristen M. Wrocklage, Brian Schweinsburg, J. Cobb Scott, Brenda Martini, Lynnette A. Averill, Steven M. Southwick, John H. Krystal, Chadi G. Abdallah

**Affiliations:** 1 National Center for PTSD—Clinical Neurosciences Division, US Department of Veterans Affairs, West Haven, CT, USA; 2 Department of Psychiatry, Yale University School of Medicine, New Haven, CT, USA; 3 Department of Psychology, Gaylord Specialty Healthcare, Wallingford, CT, USA; 4 Department of Psychiatry, Perelman School of Medicine, University of Pennsylvania, Philadelphia, PA, USA; 5 VISN4 Mental Illness Research, Education, and Clinical Center, Philadelphia VA Medical Center, PA, USA

**Keywords:** posttraumatic stress disorder, structural magnetic resonance imaging, hippocampus, amygdala, anterior hippocampus, hippocampal shape, vertex-wise analysis, shape analysis, veterans, morphometry

## Abstract

**Background:**

The hippocampus and amygdala have been repeatedly implicated in the psychopathology of posttraumatic stress disorder (PTSD). While numerous structural neuroimaging studies examined these two structures in PTSD, these analyses have largely been limited to volumetric measures. Recent advances in vertex-based neuroimaging methods have made it possible to identify specific locations of subtle morphometric changes within a structure of interest.

**Methods:**

In this cross-sectional study, we used high-resolution magnetic resonance imaging to examine the relationship between PTSD symptomatology, as measured using the Clinician Administered PTSD Scale for the DSM-IV, and structural shape of the hippocampus and amygdala using vertex-wise shape analyses in a group of combat-exposed U.S. Veterans (N = 69).

**Results:**

Following correction for multiple comparisons and controlling for age and cranial volume, we found that participants with more severe PTSD symptoms showed an indentation in the anterior half of the right hippocampus and an indentation in the dorsal region of the right amygdala (corresponding to the centromedial amygdala). Post hoc analysis using stepwise regression suggest that among PTSD symptom clusters, arousal symptoms explain most of the variance in the hippocampal abnormality, whereas reexperiencing symptoms explain most of the variance in the amygdala abnormality.

**Conclusion:**

The results provide evidence of localized abnormalities in the anterior hippocampus and centromedial amygdala in combat-exposed U.S. Veterans suffering from PTSD symptoms. This novel finding provides a more fine-grained analysis of structural abnormalities in PTSD and may be informative for understanding the neurobiology of the disorder.

## Introduction

Posttraumatic stress disorder (PTSD) is a common mental illness, with an estimated lifetime prevalence rate of 6.8% in the general population in the United States,^1^ and an estimated 23% among post-9/11 U.S. Veterans.^2^ Yet, the neurobiological mechanisms underlying the disorder are not fully understood, and the availability of effective pharmacotherapies is scarce.^3–5^ Better understanding of the underlying pathophysiology may contribute to the development of novel rational therapeutics. In this article, we investigated the relationship between PTSD symptoms and the shape (vertex-based) of two subcortical structures critical to stress response and emotion regulation that have been repeatedly implicated in the pathophysiology of PTSD:^6–9^ the hippocampus and amygdala. In contrast to the presence of numerous vertex-based cortical PTSD studies,^10–25^ structural analyses of the hippocampus and amygdala have largely been limited to volumetric measures (i.e., total or voxel-based volumes). This vertex-based approach complements previous volumetric findings and might provide enhanced localization of the structural abnormalities within the hippocampus and amygdala.

Multiple neuroimaging studies have examined brain regions involved in PTSD symptomatology in an effort to characterize the mechanisms of the disorder and ultimately inform treatment. In animal models, chronic stress has been shown to have opposing effects on synaptic plasticity in the hippocampus and the amygdala. Trauma and stress induce synaptic degeneration and neuronal atrophy in the hippocampus but result in trophic changes and synaptogenesis in the amygdala.^26^ These preclinical findings of atrophy have been paralleled by clinical evidence of reduced hippocampal volumes in PTSD patients. A large number of studies, including several meta-analyses, have reported total volume reduction in the hippocampi of PTSD patients^16,27–39^ (note that citations do not represent an exhaustive list; for further details refer to recent meta-analyses by Kühn et al.^36^ and Li et al.^37^). Nevertheless, it should be noted that a number of studies and at least one meta-analysis have failed to replicate these volumetric changes.^40–43^ In the case of the amygdala, the increased synaptogenesis in preclinical models has so far mostly been captured from the purview of functional imaging, where findings of a hyperactive amygdala are prevalent.^44^ The evidence from structural imaging is inconclusive, as both reduced^6,39,45,46^ and increased^7^ amygdala volumes have been reported. Other studies^40–42^ including one meta-analysis^47^ failed to identify amygdala volumetric abnormalities associated with PTSD.

The vast majority of structural neuroimaging studies investigating these two structures have so far made use of volume measurements, i.e., region of interest (ROI) or voxel-based morphometry (VBM). Despite their value, volumetric measurements are unable to capture abnormalities related to the shape of a structure and possess limited ability to localize abnormalities within regions of interest. Furthermore, several questions have been raised regarding the sensitivity of VBM towards subtle morphometric changes, particularly with regard to subcortical nuclei.^48–50^ The hippocampus and amygdala are heterogeneous structures with different subregions having unique cellular architectures and developmental and functional properties.^51,52^ For example, the anterior hippocampus is thought to perform stress- and emotion-related functions while the posterior hippocampus is associated with various cognitive functions.^53^ In addition, differing functional PTSD-related abnormalities have been found in the anterior versus posterior hippocampus.^9,54^ Similarly, the basolateral amygdala (BLA) is involved in sensory integration with afferents from the various sensory and association cortices; in contrast, the centromedial amygdala (CMA) mediates efferent fear response.^52,55^ In animal models, trauma and stress-induced amygdala hypertrophy has been most evident in the BLA.^56,57^ It is therefore conceivable that the inconsistent findings in the literature could be partially due to an inherent limitation in gross volumetric measurement, namely the inability to localize abnormalities within the structure in question or to detect structural changes other than total volumes. Additionally, the multifaceted phenotype observed in PTSD, which consists of dimensions of depressive as well as hyperarousal and reexperiencing symptoms,^58^ could be each linked to a particular abnormality within the hippocampus or amygdala.

Recent advances in neuroimaging methods have made it possible to identify specific locations of subtle morphometric changes within a structure of interest. This morphometric approach, known as shape analysis or vertex-wise analysis, aims to measure shape differences by analyzing surface representation rather than individual voxels.^59^ Here, we report on vertex-wise shape analyses of the hippocampus and amygdala and the relationship with PTSD symptomatology, as measured with the Clinician Administered PTSD Scale for the DSM-IV (CAPS).^60^ Rather than using a dichotomy consisting of PTSD patients and controls, we employ a single-group dimensional approach in a sample of combat-exposed U.S. Veterans to capture a continuous spectrum of PTSD symptoms for the primary analysis. We attempt to answer additional questions relating to confounds, symptom clusters, and sex differences in post hoc analyses.

## Methods

### Participants and Clinical Assessments

A total of 69 combat-exposed U.S. Veterans (aged 21–60) participated in this study. Details of the study sample and procedures were previously reported.^61^ Briefly, inclusion criteria required at least one combat tour deployment, and exclusion criteria included psychotic disorder or bipolar disorder, attention-deficit/hyperactivity disorder, learning disorder, moderate or severe traumatic brain injury (TBI), brain tumor, epilepsy or other neurological disorders, current benzodiazepine use, and magnetic resonance imaging contraindication. To ensure external validity and generalizability of the findings to the target population, the following were not considered exclusionary due to their high co-occurrence in Veterans with PTSD: depression, anxiety, substance/alcohol use disorder, and stable antidepressant regimens.

PTSD diagnosis, overall symptom severity, and symptom cluster severity (i.e., numbing-avoidance, hyperarousal, re-experiencing) were assessed using CAPS.^62^ Combat exposure severity was assessed using the Combat Exposure Scale.^63^ Depressive and anxiety symptoms were assessed using the Beck Depression Inventory^64^ and Beck Anxiety Inventory,^65^ respectively. A measure of estimated pre-exposure intellectual functioning was determined using the Wechsler Test of Adult Reading.^66^ Psychiatric comorbidities were assessed using the Structured Clinical Interview for the DSM-IV.^62^

The study was approved by Institutional Review Boards at the VA Connecticut Healthcare System and Yale University. Written informed consent was obtained from all participants.

### Neuroimaging Methods

A Siemens TIM Trio 3.0 Tesla magnet with a 32-channel head coil was used. magnetic resonance imaging acquisition included a T1-weighted MPRAGE (voxel size = 1 × 1 × 1 mm; TR = 2530 ms; TE = 2.71 ms; Flip = 7°). Shape processing for the hippocampus and amygdala was conducted using the FSL FIRST toolbox.^59^ Briefly, the processing included image reorientation, cropping, bias-field correction, nonlinear registration to standard space, FNIRT-based brain extraction, and structural segmentation. FIRST automated segmentation uses shape models constructed from manually segmented images; for technical details, refer to a detailed description of the method by Patenaude et al.^59^ In the standard space, the mesh representation of each structure and their mode parameters were then used to generate a study specific surface standard for the hippocampus and the amygdala (i.e., the average of all subjects). Per subject, each vertex anatomical location was projected onto the standard surface. The signed perpendicular distance between each projected vertex and the standard surface represent the projection values (negative = depression/indentation & positive = inflation), which were used in the study statistical analysis.

### Statistical Analyses

Vertex-wise shape for the hippocampus and amygdala was correlated with CAPS score using FSL Randomise with nonparametric permutations (number of permutations = 5000) and cluster-based thresholding (z > 2.3, corrected *α* = 0.05),^67^ controlling for age and cranial volume.

For post hoc analyses, we extracted the average of the vertices in the segment showing significant abnormality in the vertex-wise analysis as a measure of average abnormality. It is important here to note that the post hoc analyses should not be considered as independent evidence, but rather an exploratory assessment to inform future studies and meta-analyses, and to better characterize the variables associated with the abnormalities identified by the vertex-wise results. We first conducted partial correlation analyses between CAPS severity and average abnormality in the hippocampus or amygdala, covarying for each of the following putative confounds separately: sex, Major Depressive Disorder diagnosis, substance/alcohol abuse, other psychiatric diagnosis, combat exposure severity, pre-exposure intelligence, education, medication status, and TBI status. Before proceeding to test the symptom cluster scores of numbing-avoidance, hyperarousal, and re-experiencing, the variance inflation factor (VIF) was used to assess for problematic multicollinearity, by entering the three subtype scores simultaneously in a multiple regression with either the hippocampus or amygdala abnormality as dependent variables. Next, we conducted a stepwise multiple regression to assess which of the PTSD symptom clusters contribute most to the abnormalities. For each structure separately, the average abnormality was entered as a dependent variable, and the three symptom cluster scores were entered into the model (*p*-value thresholds: entry = 0.05, removal = 0.1). Considering the known sex differences in PTSD,^68,69^ we assessed whether one group was disproportionally influencing the results. Correlation analyses were used to examine the relationship between the shape abnormalities and CAPS scores for females and males separately. Finally, to facilitate the interpretation and integration of our results by other groups, we included a group comparison between Veterans with PTSD and without PTSD (non-PTSD), controlling for age and intracranial volume.

## Results

Demographic variables and clinical characteristics are presented in Table 1. On average, participants had a moderate level of PTSD symptoms, and 51% met DSM-IV criteria for PTSD.

**Table 1.** Demographic and clinical characteristics.

	Mean ± SEM or %
Age (years)	34.4 ± 1.1
Sex (% female)	11%
WTAR standard score	103 ± 1.0
Education (years)	14.0 ± 0.2
CAPS	44.7 ± 3.6
CES	18.0 ± 1.2
BDI	18.9 ± 1.5
BAI	13.4 ± 1.3
DSM-IV axis I	65%
PTSD	51%
MDD	18%
SUD	23%
Anxiety disorder	7%
Psychotropic medication	33%
Mild TBI	59%

Note: SEM: Standard Error of Means; WTAR: Wechsler Test of Adult Reading; CAPS: Clinician Administered PTSD Scale for the DSM-IV; CES: Combat Exposure Scale; BDI: Beck Depression Inventory; BAI: Beck Anxiety Inventory; PTSD: Posttraumatic Stress Disorder; MDD: Major Depressive Disorder; SUD: Substance/Alcohol Use Disorder; Anxiety: Panic Disorder, Generalized Anxiety Disorder, Obsessive Compulsive Disorder; TBI: Traumatic Brain Injury.

### Vertex-Wise Analysis

**Figure 1.**
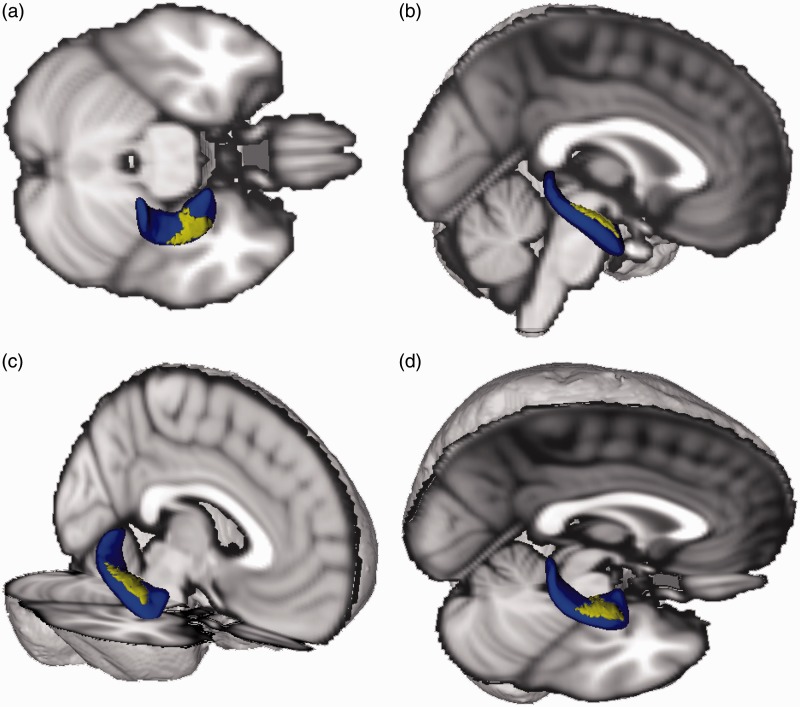
Localized right hippocampus abnormality. Three-dimensional (3D) depiction (blue) of the right hippocampus showing the location of the indentation (yellow) in participants with severe PTSD symptoms. Standard brain added to aid visualization. Superior (a), lateral (b), anterolateral (c), superolateral (d) views.

**Figure 2.**
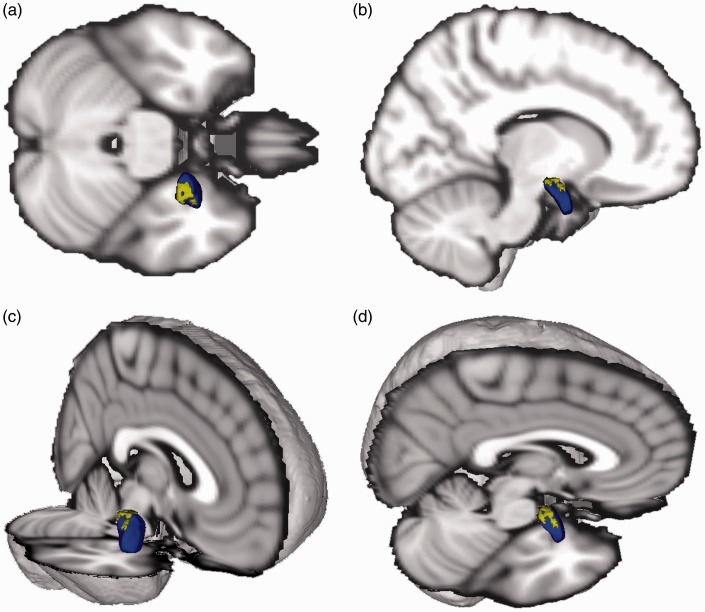
Localized right amygdala abnormality. Three-dimensional (3D) depiction (blue) of the right amygdala showing the location of the indentation (yellow) in participants with severe PTSD symptoms. Standard brain added to aid visualization. Superior (a), lateral (b), anterolateral (c), superolateral (d) views.

Following correction for multiple comparisons using Randomise and controlling for age and cranial volume, the vertex-wise analysis revealed a negative correlation between CAPS severity and hippocampal vertices, such that participants with more severe PTSD symptoms showed a depression/indentation in the anterior half of the right hippocampus (Figure 1), but no significant alterations in the posterior right hippocampus or all of the left hippocampus. Similarly, we found a negative correlation between CAPS severity and amygdala vertices, such that participants with more severe PTSD symptoms showed a depression/indentation in the dorsal region (corresponding to the CMA) of the right amygdala, but no significant alterations elsewhere in the amygdala (Figure 2).

### Post Hoc Analyses

The correlation between hippocampus/amygdala shape and CAPS remained significant (*p* < 0.05) after controlling for each of the following variables separately: age, gender, Major Depressive Disorder diagnosis, other psychiatric diagnosis, combat exposure severity, estimated pre-exposure intelligence, education, substance/alcohol abuse, medication status, and TBI.

All symptom cluster scores were significantly associated with the identified indentations in both structures (bivariate models are presented in Tables 2 and 3, respectively). The multiple regression model revealed a moderate level of multicollinearity between the symptom clusters (VIF < 4 for all cases). In the stepwise model relating to the hippocampus, initially all three terms that were entered were significantly correlated with the indentation (*p* < 0.01). Subsequently, only the hyperarousal score survived and demonstrated a significant effect, with the model explaining 12% of the variance of the abnormality (R^2 ^= 0.12; *p* = 0.02). Neither the numbing-avoidance nor re-experiencing scores were found to have significant effect in the model as a whole and were excluded (*p* > 0.4). Similarly, in the stepwise model relating to the amygdala, all three terms were significantly correlated with the indentation (*p* < 0.01), but only the re-experiencing score subsequently survived and demonstrated a significant effect, with the model explaining 15% of the amygdala abnormality variance (R^2 ^= 0.15; *p* = 0.01). Neither the numbing-avoidance nor re-experiencing scores were found to have significant effect in the model as a whole and were excluded (*p* > 0.7). A summary of the stepwise models can be found in Table 4.

**Table 2.** Bivariate correlation matrix for PTSD symptom clusters and the hippocampal abnormality.

		Right hippocampus	Re-experiencing	Hyperarousal	Numbing-avoidance
Right hippocampus	*r*	1			
	*p*	*			
Re-experiencing	*r*	−0.339	1		
	*p*	0.002	*		
Hyperarousal	*r*	−0.359	0.785	1	
	*p*	0.001	*	*	
Numbing-avoidance	*r*	−0.245	0.791	0.825	1
	*p*	0.021	*	*	*

* *p* < 0.001.

**Table 3.** Bivariate correlation matrix for PTSD symptom clusters and the amygdala abnormality.

		Right amygdala	Re-experiencing	Hyperarousal	Numbing-avoidance
Right amygdala	*r*	1			
	*p*	*			
Re-experiencing	*r*	−0.382	1		
	*p*	0.001	*		
Hyperarousal	*r*	−0.319	0.785	1	
	*p*	0.004	*	*	
Numbing-avoidance	*r*	−0.289	0.791	0.825	1
	*p*	0.008	*	*	*

* *p* < 0.001.

**Table 4.** Stepwise regression models with PTSD symptom clusters and the localized abnormalities.

Structure	Predictor	β	SE	Standardized β	*t*	*p*	R^2^
Right hippocampus	Arousal	−.041	0.013	−0.359	−3.153	0.002	0.129
Right amygdala	Re-experiencing	−.032	0.009	−0.382	−3.381	0.001	0.146

Note: The original model in both cases included CAPS numbing-avoidance, arousal, reexperiencing.

In the male group, abnormality in both structures correlated with the total CAPS score (hippocampus: *r* = −0.4, *p* = 0.02; amygdala: *r* = −0.4, *p* = 0.02), whereas in the female group, none of the two structures were found to be significantly correlated with the total CAPS score (hippocampus: *r* = 0.1, *p* = 0.9; amygdala: *r* = −0.23, *p* = 0.7). It is important to note that women only constituted 11% (8 participants) of the total sample.

Compared to Veterans with no PTSD, the abnormality in the PTSD group was as follows: hippocampus (PTSD: mean ± SEM = −0.417 ± 0.175; non-PTSD: mean ±SEM = 0.454 ± 0.183; *p* = 0.001) and amygdala (PTSD: mean ± SEM = −0.258 ± 0.121; CC: mean ± SEM =0.282 ± 0.127; *p* = 0.003).

## Discussion

Using a vertex-wise approach, we demonstrated shape abnormalities in the right anterior hippocampus and dorsal amygdala associated with increased PTSD symptom severity. In the hippocampus, the direction of the change—namely, indentation/depression—is consistent with previous volumetric literature showing shrinkage of this structure in PTSD.^16,27–35^ In the amygdala, where prior evidence in PTSD has been inconclusive, we found an indentation grossly overlapping with the CMA area, but no changes in the BLA, an area that is particularly sensitive to stress-related hypertrophy in animal models.^56,57^

The hippocampus has long been identified as an important structure in PTSD; its failure to recognize contextual cues in the absence of threat, and to relay that information to the amygdala and the vmPFC, is believed to contribute to the observed exaggerated states of fear and hyperarousal.^3^ A smaller hippocampal volume in PTSD has been widely reported in the literature^16,27–35^; however, the majority of these studies considered the hippocampus as a single entity. Evidence from animal studies demonstrating functional segmentation within the hippocampus is accumulating; according to such models, the anterior portion of the hippocampus plays a central role in stress, emotion, and affect, while the posterior portion is primarily involved in spatial memory and other cognitive functions.^53^ Converging evidence suggests a gradient along longitudinal axis of the hippocampus where coarse field representations take place anteriorly while more fine-grained representations are encoded posteriorly.^70^ It has been suggested that this “sparse” representation in the anterior hippocampus is optimized for cross-environment generalization (i.e., pattern completion); in contrast, denser and more fine-grained segments present posteriorly enable filtering out the interference, particularly useful in more similar environments (i.e., pattern separation).^70^ An altered balance between pattern completion and pattern separation is hypothesized to underline overgeneralization of fear and context-inappropriate fear response in PTSD.^71,72^ Our analyses identified an indentation in the hippocampus associated with increasing PTSD severity that is localized to the right anterior half of the hippocampus, which could be reflecting a dysfunctional dynamic between these two hippocampal functions.^9^ Reduction in the anterior but not posterior hippocampal volume has also been previously described in Veterans with PTSD.^73^ Future studies can further assess the anterior hippocampal abnormalities by examining the role of hippocampal subfields (e.g., CA3) in the pathophysiology of PTSD. Finally, it is noticeable that in the current study, the shape abnormalities were detected in the right hippocampus, whereas in several previous PTSD studies, volumetric differences were reported either bilaterally or on the left.^36^ It remains to be seen whether this observation is limited to our sample or whether our results represent a lateralization of hippocampal shape abnormalities to the right hemisphere.

The amygdala has a crucial role in fear learning and expression, as well as the detection of threat.^55^ While numerous functional neuroimaging studies have shown heightened amygdala reactivity in PTSD patients in various experimental paradigms, the evidence for amygdala structural abnormalities has been contradictory.^44^ This may have been due to the suboptimal sensitivity of the employed volumetric and VBM approaches. Using vertex-wise analyses, we identified a region of indentation in the dorsal area of the right amygdala associated with increased PTSD severity—which anatomically corresponds to the central nucleus of the amygdala (CEA). The amygdala consists of multiple nuclei with distinct roles. The CMA, which comprises the CEA and the medial nucleus, plays a major role in affect expression and anxiety-related behavior, while the BLA is involved in perception and emotional modulation.^55^ In animal models, the BLA, but not the CMA, is a site of synaptogenesis under conditions of chronic stress.^26,56,57^ It is therefore conceivable that inconsistent results in previous volumetric studies of the amygdala reflect mixed changes that could include concomitant CMA atrophy and BLA hypertrophy. While we were not able to identify regions of hypertrophy in the BLA, this approach highlights the importance of pursuing similar analyses in larger samples of PTSD patients.

Using CAPS symptom cluster scores for numbing-avoidance, hyperarousal, and re-experiencing, we probed the association between the identified abnormalities in the hippocampus and amygdala and these symptoms. The results of the stepwise analysis support a model in which PTSD arousal and re-experiencing symptoms contribute most to the variance of the localized abnormalities in the hippocampus and amygdala, respectively. Although the amygdala plays a central role in arousal,^55^ one possibility that could explain this apparent discrepancy could be related to the location of the abnormality in the CMA area. Recent evidence selectively implicates the BLA but not the CMA in the afferent aspect of arousal in functional neuroimaging studies.^74^

Women have a higher life-time risk of developing PTSD, and the manifestation of the disorder is often more pronounced.^68,69,75^ Although female participants only constituted 11% (8 participants) of our total sample, we assessed how the results of this subgroup relate to the predominantly male sample; particularly, whether in this subgroup the relationship between PTSD severity and structural abnormality would be stronger. In the female group, we failed to show a statistically significant relationship between PTSD severity and the shape abnormalities that we detected at the level of the whole sample. It is likely that this is due to small number of observations, and should not be interpreted as a confirmatory negative finding.

There are several limitations in our approach. Our study was cross-sectional, which precludes conclusions regarding causality between brain abnormalities and symptomatology. It remains to be determined whether these localized structural abnormalities represent premorbid vulnerability or trauma-related changes. Another limitation is related to our sample, which consisted of predominantly male participants, with women only comprising 11% of the total sample. This could limit the generalizability of our findings to women. Medication status as well as co-morbid psychiatric and substance abuse disorders were not excluded so that our sample was representative of the PTSD population, although this limits internal validity. Additionally, the sample size did not permit the inclusion of all 11 potential confounds in the primary analysis. Thus, we only included the two main putative confounds (age and intracranial volume), while the remaining variables were assessed in a post hoc fashion which should be interpreted with caution. All post hoc analyses were conducted on the average volume of indentation detected in the primary vertex analysis. Attempting to control for multiple potential confounds at this level would have reduced the statistical power. It is therefore important to reiterate that the post hoc analyses are not independent^76^ and were included solely for the purpose of providing preliminary data to better understand the different factors that might have contributed to these shape changes. The study strengths include the use of a validated neuroimaging approach that allows identification of morphological abnormalities beyond total volume^59^ and a dimensional approach to examine the effects of PTSD symptoms across the target population of combat-exposed U.S. Veterans.

In sum, this study makes a unique contribution to the literature by demonstrating shape abnormalities in the anterior part of the hippocampus and the dorsal amygdala, which future studies might use to further explore the neurobiology of PTSD and ultimately guide treatment development. By identifying these abnormalities, our study extends prior research on structural brain abnormalities in PTSD that can be detected using neuroimaging methods.
